# Human IL-6R^hi^TIGIT^−^ CD4^+^ CD127^low^CD25^+^ T cells display potent *in vitro* suppressive capacity and a distinct Th17 profile

**DOI:** 10.1016/j.clim.2017.03.002

**Published:** 2017-06

**Authors:** Ricardo C. Ferreira, Daniel B. Rainbow, Arcadio Rubio García, Marcin L. Pekalski, Linsey Porter, João J. Oliveira, Frank Waldron-Lynch, Linda S. Wicker, John A. Todd

**Affiliations:** a JDRF/Wellcome Trust Diabetes and Inflammation Laboratory, Wellcome Trust Centre for Human Genetics, Nuffield Department of Medicine, NIHR Oxford Biomedical Research Centre, University of Oxford, Oxford, UK; b JDRF/Wellcome Trust Diabetes and Inflammation Laboratory, Department of Medical Genetics, NIHR Cambridge Biomedical Research Centre, Cambridge Institute for Medical Research, University of Cambridge, Cambridge, UK; c Experimental Medicine and Immunotherapeutics, Department of Medicine, NIHR Cambridge Biomedical Research Centre, University of Cambridge, Cambridge, UK; d NIHR Cambridge Clinical Trial Unit, Cambridge NHS University Hospitals Trust, Cambridge Biomedical Research Centre, University of Cambridge, Cambridge, UK

## Abstract

To date many clinical studies aim to increase the number and/or fitness of CD4^+^ CD127^low^CD25^+^ regulatory T cells (Tregs) *in vivo* to harness their regulatory potential in the context of treating autoimmune disease. Here, we sought to define the phenotype and function of Tregs expressing the highest levels of IL-6 receptor (IL-6R). We have identified a population of CD4^+^ CD127^low^CD25^+^ TIGIT^−^ T cells distinguished by their elevated IL-6R expression that lacked expression of HELIOS, showed higher CTLA-4 expression, and displayed increased suppressive capacity compared to IL-6R^hi^TIGIT^+^ Tregs. IL-6R^hi^TIGIT^−^ CD127^low^CD25^+^ T cells contained a majority of cells demethylated at *FOXP3* and displayed a Th17 transcriptional signature, including *RORC* (RORγt) and the capacity of producing both pro- and anti-inflammatory cytokines, such as IL-17, IL-22 and IL-10. We propose that *in vivo*, in the presence of IL-6-associated inflammation, the suppressive function of CD4^+^ CD127^low^CD25^+^ FOXP3^+^ IL-6R^hi^TIGIT^−^ T cells is temporarily disarmed allowing further activation of the effector functions and potential pathogenic tissue damage.

## Highlights

• IL-6R is highly expressed in certain Treg subsets.
• IL-6R^hi^TIGIT^−^ CD127^low^CD25^+^ T cells contain a subset of antigen-experienced Tregs with potent suppression capacity.
• IL-6R^hi^TIGIT^−^ Tregs display a Th17 transcriptional profile *ex vivo*, and the capacity to migrate to the gut.
• IL-2 treatment in humans elicits the trafficking and expansion of Tregs in circulation.

## 1 Introduction

CD4^+^ CD127^low^CD25^+^ regulatory T cells (Tregs), the majority of which differentiate in, and egress from, the thymus are an immune subset with a critical role in the maintenance of self-tolerance and protection against tissue damage and autoimmunity [1]. This Treg subset is defined by the expression of the transcription factor FOXP3, which is essential for their differentiation, maintenance and function [2]. The stable FOXP3 expression that is required for Treg function depends on demethylation of a sequence in the first intron of the gene, termed the Treg-specific demethylated region (TSDR) [3]. FOXP3, along with other transcription factors, suppresses the expression of the IL-2 gene in these Tregs [2], [4], making them critically dependent on IL-2 production from conventional or effector T cells (Teffs). For example, reduction of IL-2 in the pancreatic islets leads to a loss of FOXP3 expression and Treg numbers in a mouse model of autoimmune type 1 diabetes (T1D) [5], [6]. Furthermore, in humans, variation of genes in the IL-2 pathway, for example *IL2RA* encoding CD25, alters susceptibility to autoimmune diseases, including T1D [7], [8], [9], [10]. The leading disease-associated variant of *IL2RA* is correlated with reduced expression of CD25, leading to a decrease in Treg IL-2 sensitivity and suppressive ability [11], [12].

These findings have provided the rationale for clinical studies aiming at expanding Tregs *in vitro*, or increasing Treg numbers and fitness *in vivo* by IL-2 administration, for the treatment of autoimmune diseases [13], [14], [15]. However, there is growing evidence of heterogeneity and plasticity among Tregs [16], [17], [18]. A population of FOXP3^+^ Tregs has been identified in humans that constitutively expresses the Th17 lineage-specific transcription factor RORγt, a portion of which produce IL-17 upon *in vitro* activation [19], [20], [21], [22]. More recently, two studies have demonstrated that the frequency of mouse RORγt^+^ FOXP3^+^ Th17 Tregs in the colon is determined by the composition of the intestinal commensal microbiota [23], [24]. These data indicate that Th17 Tregs play a key role in the regulation of the intestinal Th17 immune responses to commensal and pathogenic bacteria, while preventing inflammation. Consistent with this hypothesis, adoptive transfer of RORγt^+^ FOXP3^+^ Th17 Tregs completely abrogated the development of inflammation in a mouse colitis model, and displayed increased suppressive capacity *in vivo* compared to conventional RORγt^−^ FOXP3^+^ Tregs [25]. Further supporting the suppressive function of these cells *in vivo*, another study also showed that a subset of FOXP3^int^RORγt^+^ Tregs were able to prevent the development of autoimmune diabetes in the NOD model [26]. The mutually opposing regulatory functions of the FOXP3 and RORγt transcription factors have raised questions regarding the origin and differentiation of Th17 Tregs, leading to the hypothesis that Th17 T effector cells (Teff) and Tregs could share a similar differentiation pathway, and have been selected to ensure immunity at the barrier surfaces and the adaptation of the host to commensal bacteria [27].

IL-6, which is a potent driver of inflammatory disease, is instrumental for the commitment to the Th17 lineage, by inhibiting FOXP3-mediated suppression of RORγt, thereby promoting the differentiation of the Th17 Teff lineage in mice [28], [29], [30]. In addition, studies in mice have also shown that IL-6 signalling is an established factor to inhibit the suppressive function of Tregs [31], although this effect has been shown to be Treg extrinsic, through the activation of Teffs, which are able to escape Treg suppression [32]. Furthermore, in the presence of IL-6, Tregs have been reported to induce IL-17 production by Th17 Teffs cells and have been shown to be themselves capable of producing IL-17 [33].

The effects of IL-6 in studies of human CD4^+^ T cells differ from those reported using cells from mice in several aspects. For example, IL-6 does not contribute to the differentiation of naïve CD4^+^ T cells to the Th17 lineage as it can in mice, although IL-1β and IL-6 together induce IL-17A secretion from human memory CD4^+^ Th17 cells [34]. Furthermore, the exact role of IL-6 signalling on human Treg function remains uncertain. In psoriasis patients, elevated IL-6 production in affected skin has been suggested to promote the differentiation of IL-17-producing pathogenic Th17 cells and reduce the suppressive capacity of Tregs, although this latter effect is likely to be indirect [35]. Evidence from Bhela *et al*. also supports the hypothesis that Tregs are not the direct target of IL-6 for its ability to elicit resistance to Treg-mediated suppression [36]. In contrast, Bending *et al*. have reported that IL-6 provides pro-regulatory signals directly to human Tregs [37].

Previously, a genetic variant in *IL6R* (rs2228145 A > C; Asp358Ala) has been associated with human inflammatory diseases, including T1D [38], ankylosing spondylitis [39] and rheumatoid arthritis [40], presumably due to a greater expression of the IL-6 receptor and therefore a higher IL-6 signalling capacity in CD4^+^ Teffs expressing the common Asp358 susceptibility allele compared to Ala358 [38]. Furthermore, IL-6 signalling is increased in T1D patients owing to increased IL-6R expression [41]. These results indicate that inhibition of IL-6 signalling could be a therapeutic approach in T1D, as it is in other human inflammatory diseases [42], [43]. Nevertheless, blockade of IL-6, which is a key pathway in immune defence, could raise the risk of serious infections in T1D patients and hence targeting the therapy to a specific cell type could offer advantages in therapy. In the current study we show that a portion of the memory IL-6R^hi^ CD127^low^CD25^+^ T cells (here designated as Tregs) lack TIGIT and HELIOS expression and have a constitutively elevated expression of the effector Treg marker CTLA-4, and contain a heterogeneous group of cells that as a total population are highly suppressive, with portions expressing a Th17 signature, a demethylated *FOXP3* TSDR and the FOXP3 transcription factor protein. IL-6R^hi^ TIGIT^−^ Tregs contain a subset of cells having a tissue-homing chemokine receptor profile analogous to the intestinal FOXP3^+^ RORγt^+^ Th17 Treg subset recently characterised in mice [23], [24], [25]. We also show that, similarly to other Treg subsets, circulating IL-6R^hi^ Tregs were expanded in number after administration of IL-2. These data indicate that IL-6R^hi^ TIGIT^−^ Tregs will be highly sensitive to the endogenous production of IL-2 induced by infection or inflammation, thereby stimulating the recruitment of these highly suppressive Tregs, which we suggest could be normally resident in the intestine and mesenteric lymph nodes, to sites of infected cells or inflammation, where these Tregs curtail effector activities and prevent potentially pathogenic tissue damage. However, in the presence of high tissue concentrations of IL-6 these cells might lose their suppressive capacity temporarily to help fight infection, an effect that would not be advantageous in a site of chronic inflammation in which a pathogen was not present. With the combination of surface markers we have defined here, these cells can now be purified from whole blood and biopsy tissues to further study their functions and their putative immunotherapeutic properties.

## 2 Materials and methods

### 2.1 Subjects and study design

Patient selection and the protocol for the “*Adaptive study of IL-2 dose on regulatory T cells in type 1 diabetes*” (DILT1D) has been published previously [15], [44]. A subset of 22 T1D patients (median age = 26, range 18–48) were selected for this study, and assessed for the expression of IL-6R on Tregs. A blood sample was taken before treatment to establish baseline Treg frequencies and phenotypes, followed by subcutaneous administration of a single dose of recombinant human IL-2 (Proleukin/aldesleukin; dose range 45,000–737,000 IU/m^2^) on day 0. The patients were bled 90 min after treatment, and then daily to day 4 and at days 7, 9, 14, 21 and 60. The DILT1D data from individuals prior to normalisation as a group are available, however they cannot be anonymised sufficiently to be able to put into the public domain without risk of participant identification. Data are available on request, through the Cambridge University institutional repository (DOI link: https://doi.org/10.17863/CAM.832).

Study participants for all further immunophenotyping and functional assays included in this study were adult healthy volunteers recruited from the Cambridge BioResource (http://www.cambridgebioresource.org.uk/). All samples were collected after approval from the relevant research ethics committees, and written informed consent was obtained from the participants.

### 2.2 Flow cytometry

For the clinical trial participants, 30 ml whole blood were collected into lithium heparin tubes and processed within 4 h of phlebotomy. Immunostaining was performed in whole blood with specific fluorochrome-conjugated antibodies (Table 1 in Ref. [45]) at room temperature for 45 min. IL-6R expression was assessed using a phycoerythrin (PE)-conjugated antibody, which provided the better resolution in our flow cytometric setting. This was critical to increase the sensitivity of the assay, and assess quantitative differences in IL-6R expression in different T cell subsets.

Treg immunophenotyping in healthy donors was performed in fresh peripheral blood mononuclear cells (PBMCs) isolated by Ficoll gradient centrifugation (Lymphoprep; STEMCELL Technologies) from whole blood within 2 h of phlebotomy. Cells were stained with fluorochrome-conjugated antibodies against surface receptors (Table 1 in Ref. [45]) for 45 min at 4 °C. Fixation and permeabilisation was performed using FOXP3 Fix/Perm Buffer Set (BioLegend) and cells were then stained with the respective intracellular antibodies for 45 min at room temperature (Table 1 in Ref. [45]).

### 2.3 Cytokine secretion assays

To assess cytokine production, CD4^+^ T cells were isolated from whole blood by negative selection using RosetteSep (STEMCELL Technologies) within 2 h of phlebotomy. Cells were resuspended in X-Vivo15 (Lonza) + 5% heat-inactivated, filtered human AB serum (Sigma), and cultured (1–2 × 10^6^ CD4s/well) in a 24-well flat-bottom cell culture plate (CELLSTAR, Greiner) at 37 °C in the presence or absence of the 1 × Cell Stimulation Cocktail (eBiosiences), containing phorbol myristate acetate (PMA), ionomycin, and protein transport inhibitors (brefeldin A and monensin).

After 6 h culture, cells were harvested and immunostained with surface and intracellular antibodies (Table 1 in Ref. [45]). The unstimulated cells were used to determine background levels of cytokine production. Dead-cell exclusion was performed using the eFluor780 Fixable Viability Dye (eBiosciences).

### 2.4 Intracellular pSTAT3 immunostainings

PBMCs were isolated from three healthy donors by Ficoll gradient centrifugation from whole blood within 2 h of phlebotomy. IL-6 sensitivity of the memory Treg and Teff subsets was determined by intracellular pSTAT3 immunostaining in freshly isolated PBMCs in response to IL-6 stimulation *in vitro*, as previously described [38].

### 2.5 Cell sorting

Cell sorting was performed using a BD FACSAria Fusion flow cytometer (BD Biosciences) after pre-enrichment of CD4^+^ T cells from whole blood by negative selection. Fluorescence-conjugated antibodies used for sorting are described in Table 1 in Ref. [45]. Sorting efficiencies were determined in four donors, based on IL-6R and TIGIT expression and ranged between 90 and 99%.

### 2.6 *FOXP3* and *CTLA4* demethylation assays

Epigenetic profiling of the *FOXP3* TSDR and the CTLA-4 gene was performed using a next-generation sequencing method described previously [46]. DNA was extracted from flow sorted cells from three independent healthy male donors (containing a single copy of the FOXP3 gene on chromosome X) and bisulfite treated using Epitect fast lyse all kit (Qiagen). Bisulfite treated DNA samples were used as the template for first round PCR of the *FOXP3* TSDR (ChrX: 49,260,689–49,260,861) and *CTLA4* exon 2 locus (Chr2: 203,870,594–203,870,872). A second round PCR was performed to add a bar code sequence, before samples were pooled and sequenced on an Illumina MiSeq.

### 2.7 *In vitro* proliferation and suppression assays

To assess the suppressive capacity of IL-6R^hi^TIGIT^−^, IL-6R^hi^TIGIT^+^ and IL-6R^lo^TIGIT^+^ Tregs, 10^4^ sorted CD45RA^−^ CD127^+^ CD25^−^ Teffs were labelled with eFluor450 Cell Proliferation Dye (eBioscience), and co-cultured with each Treg subset at various ratios (Treg:Teff 1:2 to 1:16) in X-Vivo15 + 5% human AB serum. Co-cultures were incubated for 84 h at 37 °C in V-bottom 96-well cell culture plates (CELLSTAR, Greiner) in the presence of α-CD3/CD28 activation beads (Life Technologies), at a 1:20 bead:Teffs ratio. Proliferation of the responder cells was assessed by the dilution of the proliferation dye by flow cytometry.

For the proliferation assays, sorted cells were labelled with eFluor450 Cell Proliferation Dye, and cultured in the presence of exogenous IL-2 (100 U/ml; Proleukin) and α-CD3/CD28 activation beads, at a 1:1 bead:Teff ratio.

Proliferation and suppressive capacity were calculated using the Division Index (DI) in FlowJo (Tree Star), setting 0% suppression as the condition with the respective Teffs cultured in the absence of Tregs. The DI represents the average number of cell divisions that each seeding Teff cell has undergone and was obtained using the following equation: DI = Total number of Cell Divisions/Initial number of Teff cells in culture = $\left({\left(\frac{G_{1}}{2}\right)}^{*}1+{\left(\frac{G_{2}}{4}\right)}^{*}2+{\left(\frac{G_{3}}{8}\right)}^{*}3+{\left(\frac{G_{4}}{16}\right)}^{*}4+\ldots +{\left(\frac{G_{n}}{n^{*}2}\right)}^{*}n\;\right)/\left(G_{0}+\left(\frac{G_{1}}{2}\right)+\left(\frac{G_{2}}{4}\right)+\left(\frac{G_{3}}{8}\right)+\left(\frac{G_{4}}{16}\right)+\ldots +\left(\frac{G_{n}}{n^{*}2}\right)\right)$, where *n* represents the number of divisions and *G*_(*n*)_ represents the number of cells that have undergone *n* divisions. The suppressive capacity of each experimental condition was obtained using the following equation: %Suppression = 100 − [(DI_x_/DI_0_)*100], where DI_x_ represents the Division Index of the tested experimental condition and DI_0_ represents the Division Index of the control sample with no Tregs in culture.

### 2.8 Transcriptional profiling of the Treg subsets

Gene expression profiling was performed by NanoString, using the pre-designed nCounter Human Immunology v2 Panel (NanoString Technologies). The four assessed immune cell subsets were flow sorted as described above, and 25,000 cells were collected into RLT lysis buffer (Qiagen) either: (i) directly *ex vivo*; or (ii) following *in vitro* stimulation for 165 min in the presence or absence of 50 ng/ml PMA (Sigma-Aldrich) and 500 ng/ml ionomycin (Sigma-Aldrich), without addition of protein transport inhibitors. RNA from the flow-sorted T cell subsets was extracted using the RNAeasy Micro Plus kit (Qiagen), with gDNA cleanup, following manufacturer's instructions. Total RNA samples were then hybridised to the NanoString CodeSets, following manufacturer's instructions. Expression levels were assessed using an nCounter Flex instrument (NanoString Technologies). Data were processed using the nSolver Analysis Software following normalisation of the raw read counts to the geometric mean of positive control spike-ins, and the gene expression of 15 selected housekeeping genes (*ATG10*, *C14orf166*, *CD3E*, *CD46*, *G6PD*, *GPI*, *POLR1B*, *POLR2A*, *PSMB5*, *PSMB10*, *PTPRC*, *SDHA*, *SKI*, *TOLLIP* and *TUBB*) that were found to have low variability on both the samples collected *ex vivo* and following *in vitro* stimulation.

### 2.9 Statistical analyses

Statistical analyses were performed using Prism software (GraphPad) and R (www.r-project.org.com). Statistical significance was assessed using a two-tailed non-parametric Mann-Whitney test. Comparison of immune phenotypes between the assessed Treg subsets from the same individual was performed using a two-tailed paired non-parametric Wilcoxon signed rank test.

Differential expression of normalised NanoString transcriptional data was calculated using a paired analysis with DESeq2 v1.12.3 [47], with pre-set size factors equal to one for all samples. Adjusted *P* values correspond to the false discovery rates (FDR) for differential expression, computed after correcting *P* values for multiple testing. A missing FDR is reported for genes that were found to contain an expression outlier by DESeq2 Cook's distance-based flagging of *P* values, and thus excluded from multiple testing.

## 3 Results

### 3.1 Frequency of IL-6R^hi^ Tregs is transiently increased by a single dose of IL-2 *in vivo*

To investigate the effect of IL-2 on the expression of IL-6R on Tregs *in vivo*, we performed a detailed immunophenotyping of the Treg compartment in 22 T1D patients following treatment with a single dose of IL-2. Although we acknowledge the limitation of defining Tregs with the use of surface markers and the fact that human CD4^+^ CD127^low^CD25^+^ T cells represent a heterogeneous population containing a subset of activated Teff cells, for consistency, we here refer to CD4^+^ CD127^low^CD25^+^ T cells as ‘Tregs’, as these represent the canonical surface markers used for the isolation of human Tregs for functional studies requiring viable cells. We defined in this study IL-6R^hi^ or IL-6R^lo^ Tregs, as the subset of total CD127^low^CD25^+^ CD4^+^ T cells in the upper or lower 20th percentile of the IL-6R mean fluorescent intensity (MFI) distribution, respectively (Fig. 1A in Ref. [45]). The IL-6R^hi^ gate was defined in each patient at the first visit, before IL-2 treatment, and applied to every subsequent visit. We observed clear differences in IL-6R expression in CD45RA^−^ (memory) and CD45RA^+^ (naïve) Tregs, resulting in an increased frequency of IL-6R^hi^ Tregs within memory (30.2%) compared to naïve Tregs (2.1%; Fig. 1B in Ref. [45]). The expression of IL-6R on the surface of memory Tregs translated into a similar sensitivity to IL-6 signalling *in vitro* compared to memory Teffs, as assessed by the intracellular levels of pSTAT3 in response to IL-6 stimulation (Fig. 2A–C in Ref. [45]).

**Fig. 1:**
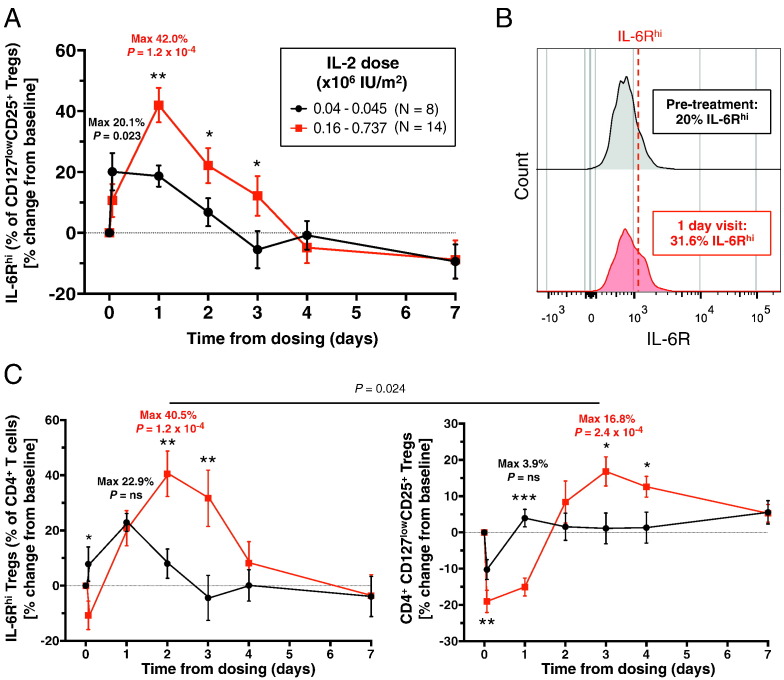
Single dose of IL-2 transiently increases the frequency of IL-6R^hi^ Tregs *in vivo*. (A) Data depict the variation (Mean ± SEM) of the frequency of IL-6R^hi^ CD127^low^CD25^+^ Tregs at each visit following IL-2 treatment compared to pre-treatment baseline (median = 20.0%; range: 19.0–21.1%) in 22 T1D patients enrolled in the “*Adaptive study of IL-2 dose on regulatory T cells in type 1 diabetes*” (DILT1D). The IL-6R^hi^ mean fluorescence intensity (MFI) threshold was defined on each donor at the first pre-treatment timepoints as the upper 20th percentile of the IL-6R MFI distribution in total CD127^low^CD25^+^ Tregs, and applied to each subsequent visit. Patients were stratified according to whether they received (i) the lower IL-2 doses of 0.04–0.045 × 10^6^ U/ml (*N* = 8; depicted in black); or (ii) the higher IL-2 doses of 0.16–0.737 × 10^6^ U/ml (*N* = 14; depicted in red). (B) Histograms depict an illustrative example of the IL-2-induced increase (58%) in the frequency of IL-6R^hi^ Tregs after 24 h of treatment with a single dose of 0.445 × 10^6^ U/ml IL-2, compared to the pre-treatment baseline. (C) Data depict the variation (Mean ± SEM) of the frequency of (i) IL-6R^hi^ Tregs (left panel) and (ii) CD127^+^ CD25^−^ Tregs (right panel) among total CD4^+^ T cells following IL-2 treatment in the same cohort of patients. Median pre-treatment baseline frequencies were 1.45% (range: 0.75–2.08%) and 7.75% (range: 3.96–10.64%) for CD4^+^ IL-6R^hi^ Tregs and total CD4^+^ CD127^low^CD25^+^ Tregs, respectively. The maximum increases over the baseline pre-treatment frequencies achieved during the course of the study are indicated for each IL-2 dosing group. *P* values for the maximum increase in the frequency of the assessed parameter in response to a single dose of IL-2 was calculated using a two-tailed paired non-parametric Wilcoxon signed rank test comparing the frequencies observed at the timepoint where the maximal increase was achieved with the respective baseline pre-treatment frequencies. *P* values for the IL-2 dose-dependent effects were calculated using a two-tailed non-parametric Mann-Whitney test comparing the frequency of IL-6R^hi^ cells between the two dose groups at each timepoint. *P < 0.05; **P < 0.01; ns = not significant.

We found that single doses of IL-2 ranging from 0.16 × 10^6^ to 0.735 × 10^6^ IU/m^2^ induced a 42.0% increase (*P* = 7.4 × 10^− 6^; Fig. 1A) in the frequency of IL-6R^hi^ Tregs 24 h after treatment; Fig. 1A, B). This effect was dose dependent, with patients receiving lower doses of IL-2 (0.04 × 10^6^–0.45 × 10^6^ IU/m^2^) showing only an 18.7% increase in the frequency of IL-6R^hi^ Tregs (*P* = 4.9 × 10^− 3^; Fig. 1A) at 24 h post-treatment. Nevertheless, we note that even in patients treated with the lower IL-2 dose, there was a significant increase in the frequency of IL-6R^hi^ Tregs (maximum increase = 20.1%, *P* = 0.023; Fig. 1A), indicating that IL-6R^hi^ Tregs are extremely sensitive to IL-2. This response to IL-2 was not restricted to the IL-6R^hi^ Treg compartment (Fig. 1C), and was consistent with the expansion of total CD4^+^ CD127^low^CD25^+^ Tregs reported previously [15]. The IL-2-induced increase in IL-6R^hi^ Treg frequency was sustained for up to three days after treatment in patients receiving the higher doses (Fig. 1A), before returning to the pre-treatment or baseline frequencies. Consistent with the previously reported decreased frequency of Tregs out of total CD4^+^ T cells in circulation 90 min post-treatment [15], we also observed a 10.8% reduction of the total number of CD4^+^ IL-6R^hi^ Tregs immediately after treatment (Fig. 1C). This reduction was less pronounced than in total CD4^+^ CD127^low^CD25^+^ Tregs (19.0%). The dose-dependent increase of IL-6R^hi^ cells induced by IL-2 was restricted to Tregs, and was not observed in memory Teffs, or in naïve T cells (Fig. 3A, B in Ref. [45]), at least at the doses of IL-2 analysed. In addition to the increased frequency of IL-6R^hi^ Tregs, we also found that IL-2 treatment induced a similar dose-dependent increase in the surface expression of IL-6R expression on Tregs at the MFI level (5.5% and 13.5% increase in the low-dose and high-dose groups, respectively, at 24 h post-treatment, *P* = 3.8 × 10^− 3^; Fig. 3C in Ref. [45]), which would lead to an increased sensitivity to IL-6.

### 3.2 Elevated IL-6R expression defines a population of antigen-experienced CD127^low^CD25^+^ Tregs

**Fig. 2:**
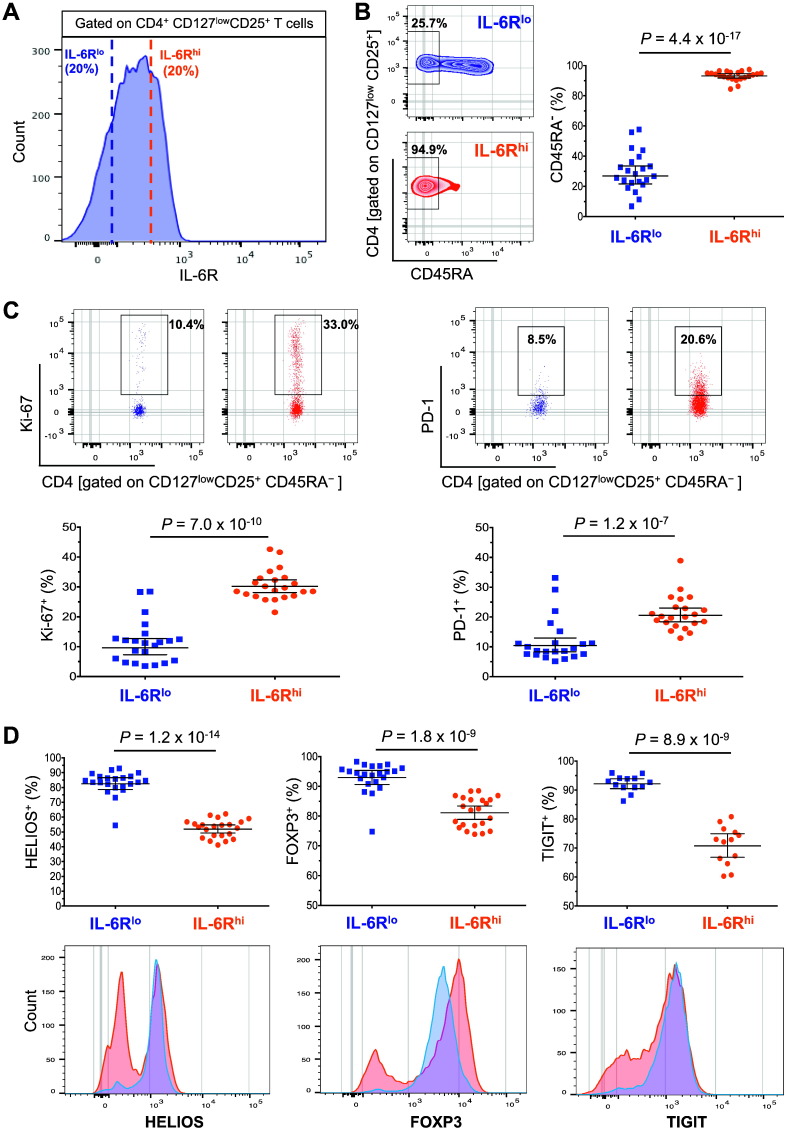
IL-6R^hi^ Tregs are activated antigen-experienced cells and show reduced expression of FOXP3, HELIOS and CD25. (A) Gating strategy for the delineation of circulating IL-6R^lo^ (depicted in blue) and IL-6R^hi^ (depicted in red) Tregs in healthy donors (*N* = 22). (B) Plot depicts the frequency (GeoMean ± 95% CI) of the CD45RA^−^ memory compartment in IL-6R^lo^ and IL-6R^hi^ Tregs. (C) Data shown depicts the frequencies (GeoMean ± 95% CI) of the proliferation marker Ki-67 and the activation marker PD-1 in CD45RA^−^ IL-6R^lo^ and IL-6R^hi^ mTregs. (D) Data depict the frequency of three Treg markers, HELIOS, FOXP3 and TIGIT in CD45RA^−^ IL-6R^lo^ and IL-6R^hi^ mTregs. Histograms depict the distribution of the Mean Fluorescence Intensity (MFI) of the assessed markers in the two subsets from one illustrative donor. *P* values were calculated using a two-tailed paired non-parametric Wilcoxon signed rank test, comparing the frequency of the assessed immune phenotypes between the IL-6R^lo^ and IL-6R^hi^ Treg subsets.

To obtain further insight into the phenotype and putative function of IL-6R^hi^ Tregs, we immunophenotyped the CD4^+^ CD127^low^CD25^+^ T cell compartment using freshly isolated PBMCs from 22 healthy donors (Fig. 2A). The first distinctive feature was that virtually all IL-6R^hi^ Tregs were of the CD45RA^−^ memory phenotype (93.2%). This was in contrast with IL-6R^lo^ Tregs (26.9%, *P* = 4.4 × 10^− 17^; Fig. 2B), indicating that IL-6R^hi^ Tregs have been previously activated in tissues in response to antigen. Given this bias in the frequency of CD45RA^−^ memory cells, for all further assays performed in this study, we normalised the assessed T cell subsets to the CD45RA^−^ memory compartment to compare the phenotype and function of cells. Memory CD45RA^−^ IL-6R^hi^ CD127^low^CD25^+^ T cells (henceforth designated as mTregs) also showed increased proliferative activity compared with their CD45RA^−^ IL-6R^lo^ counterparts, as assessed by the frequency of Ki-67^+^ cells (30.2% *versus* 9.7%, *P* = 7.0 × 10^− 10^), and increased frequency of the activation marker PD-1 (20.6% *versus* 10.4%, *P* = 1.2 × 10^− 7^; Fig. 2C), indicating that the IL-6R^hi^ cells have been activated more recently.

The IL-6R^hi^ mTreg population has more cells that are not bone-fide Tregs than the IL-6R^lo^ subset since a higher proportion lacks FOXP3 expression (81.1% FOXP3^+^ *versus* 92.9%, *P* = 1.8 × 10^− 9^). In addition, IL-6R^hi^ mTregs also showed decreased frequencies of two other markers associated with Tregs: HELIOS (51.9% *versus* 82.5%, *P* = 1.2 × 10^− 14^) and TIGIT (70.8% *versus* 92.2%, *P* = 8.9 × 10^− 9^; Fig. 2D), indicating that there is more heterogeneity among the IL-6R^hi^ mTregs as compared to IL-6R^lo^ mTregs. Although the frequency of cells expressing these Treg markers is decreased in IL-6R^hi^ mTregs, we note that their expression levels are very comparable on a per cell basis on the positive fraction of these markers on both the IL-6R^hi^ and IL-6R^lo^ mTregs, as assessed by their MFI levels (Fig. 2D).

### 3.3 Stratification of the IL-6R^hi^Treg subset by TIGIT expression

**Fig. 3:**
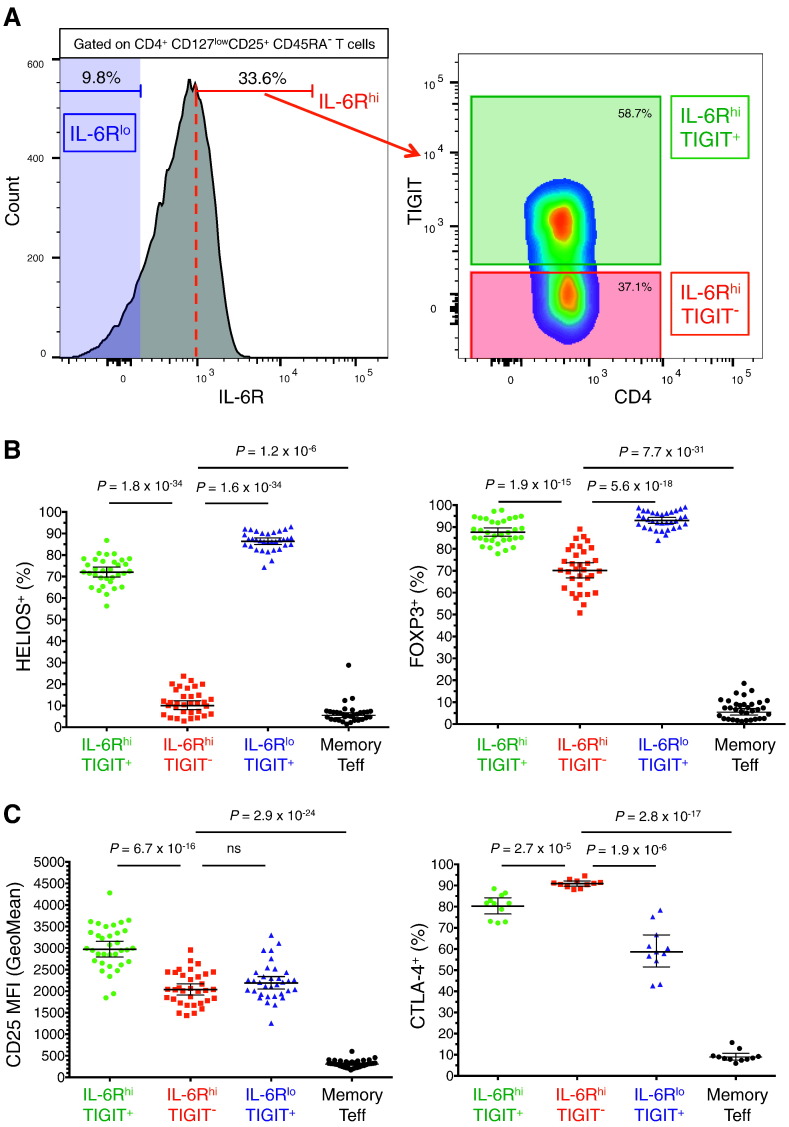
Reduction of Treg markers is restricted to the IL-6R^hi^TIGIT^−^ subset. (A) Gating strategy for the delineation of the three assessed mTreg subsets: IL-6R^lo^ (depicted in blue), IL-6R^hi^TIGIT^+^ (depicted in green) and IL-6R^hi^TIGIT^−^ (depicted in red). (B, C) Frequencies within the Treg subsets (GeoMean ± 95% CI) of the Treg markers HELIOS and FOXP3 (B), and CD25 and CTLA-4 (C) were assessed by flow cytometry in freshly isolated PBMCs from 33 healthy donors. *P* values were calculated using a two-tailed paired non-parametric Wilcoxon signed rank test.

To address the heterogeneity in IL-6R^hi^ mTregs, we stratified this subset based on the expression of the surface-expressed co-inhibitory receptor TIGIT (Fig. 3A). We observed a differential expression of IL-6R between the two TIGIT-defined mTreg subsets, with an increased frequency of IL-6R^hi^ cells in the TIGIT^−^ (45.6%), compared to TIGIT^+^ mTregs (24.3%; Fig. 1C in Ref. [45]). In contrast to IL-6R^hi^ mTregs, the vast majority of IL-6R^lo^ mTregs were TIGIT^+^ (92.2%; Fig. 2D), and therefore are described henceforth as a subset of TIGIT^+^ mTregs.

We found that the observed reduction of HELIOS^+^ and FOXP3^+^ cells within IL-6R^hi^ mTregs was restricted to the TIGIT^−^ subset, and was particularly pronounced for the HELIOS transcription factor. IL-6R^hi^TIGIT^−^ mTregs showed a markedly lower frequency of HELIOS^+^ cells (10.0%), compared to both IL-6R^hi^TIGIT^+^ (72.0%, *P* = 1.8 × 10^− 34^) and IL-6R^lo^TIGIT^+^ mTregs (86.4%, *P* = 1.6 × 10^− 34^; Fig. 3B). Similarly, IL-6R^hi^TIGIT^−^ mTregs showed a reduction in the frequency of FOXP3^+^ cells (70.1%) compared to both IL-6R^hi^TIGIT^+^ (87.7%, *P* = 1.9 × 10^− 15^) and IL-6R^lo^TIGIT^+^ mTregs (93.0%, *P* = 5.6 × 10^− 18^; Fig. 3B). Although the majority of IL-6R^hi^TIGIT^−^ mTregs were FOXP3 protein-positive, we note that a significant fraction (29.9%, range: 10.9%–49.2%; Fig. 3B) of this subset is FOXP3^−^, and therefore cannot be considered as conventional Tregs.

IL-6R^hi^TIGIT^−^ mTregs also showed some reduction in the surface expression of the IL-2 receptor (CD25) compared to their TIGIT^+^ counterparts (CD25 MFI = 2036 *versus* 2969, respectively, corresponding to a 31.4% reduction of CD25 expression; *P* = 6.7 × 10^− 16^), but not compared with IL-6R^lo^TIGIT^+^ mTregs (CD25 MFI = 2189; Fig. 3C) and still markedly higher than memory Teffs (Fig. 3B,C). In addition, IL-6R^hi^TIGIT^−^ mTregs were found to be almost completely positive for CTLA-4 (90.9%), which is a key mediator of Treg suppressive function. This frequency was higher than IL-6R^hi^TIGIT^+^ mTregs (80.3%, *P* = 2.7 × 10^− 5^) and particularly pronounced when compared to the IL-6R^lo^TIGIT^+^ subset (58.6%, *P* = 1.9 × 10^− 6^; Fig. 3C), suggesting that IL-6R^hi^TIGIT^−^ mTregs could also be suppressive *in vivo*.

### 3.4 IL-6R^hi^TIGIT^−^ Tregs are highly suppressive *in vitro* and a majority are demethylated at the *FOXP3* TSDR

**Fig. 4:**
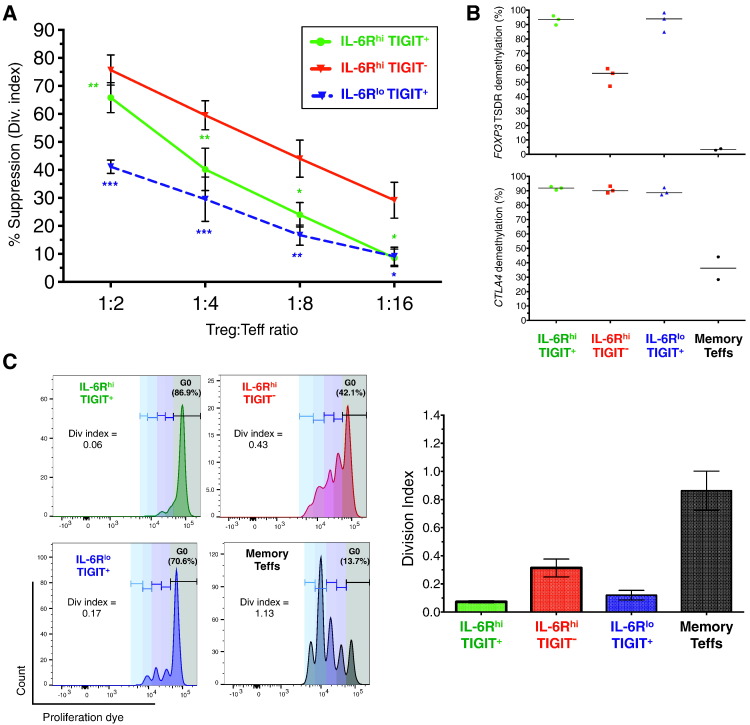
IL-6R^hi^TIGIT^−^ mTregs are highly suppressive *in vitro* and display a Treg epigenetic profile. (A) Suppressive capacity of the three mTreg subsets was assessed by the ability to supress the proliferation of autologous CD45RA^−^ Teff cells *in vitro*. Data shown depict the suppressive capacity (mean ± SEM) of the assessed mTreg subsets at diluting Treg:Teff ratios, and was obtained from sorted cells from six independent donors. *P* values were calculated using a two-tailed paired *t*-test comparing the suppressive capacity of IL-6R^hi^TIGIT^−^ to the IL-6R^lo^TIGIT^+^ and IL-6R^hi^TIGIT^+^ counterparts. *P < 0.05; **P < 0.01; ***P < 0.001. (B) Data depict the frequency of reads demethylated at eight or nine of the nine interrogated CpG sites in the *FOXP3* Treg-specific demethylation region (TSDR) or at seven out of seven CpG sites in the *CTLA4* locus. The data were obtained from sorted cells from three independent healthy male donors. Horizontal bars depict the median of the demethylated reads in each group. (C) Proliferative capacity of sorted (i) IL-6R^hi^TIGIT^+^ (depicted in green), (ii) IL-6R^hi^TIGIT^−^ (depicted in red), (iii) IL-6R^lo^TIGIT^+^ (depicted in blue) mTregs, and (iv) CD127^+^ CD25^−^ CD45RA^−^ Teff cells (depicted in black) was assessed in response to *in vitro* stimulation with α-CD3/CD28 beads and 100 U/ml exogenous IL-2. Data (mean ± SEM) were obtained from cells sorted from three independent donors. Proliferation and suppressive capacity were calculated using the Division Index in FlowJo, setting 0% suppression as the condition with the respective Teffs cultured in the absence of Tregs.

Flow-sorted purified IL-6R^hi^TIGIT^−^ mTregs suppressed the proliferation of autologous memory Teff cells *in vitro* (Fig. 4A). We observed a linear titration of the suppressive capacity of the three Treg subsets with decreasing Treg:Teff ratios, with IL-6R^hi^TIGIT^−^ Tregs showing higher suppressive capacity than conventional IL-6R^lo^TIGIT^+^ Tregs, and even higher suppressive capacity than IL-6R^hi^TIGIT^+^ mTregs (Fig. 4A). The proportion of the IL-6R^hi^TIGIT^−^ mTreg population demethylated at the *FOXP3* TSDR (54.2%), which is a hallmark of Tregs that maintain stable *FOXP3* expression, was less than the proportion expressing FOXP3 protein (70.1%; Fig. 3B). The remainder were methylated at *FOXP3*, which is in contrast with the other two conventional Treg subsets, in which nearly all of the cells (> 92%) were demethylated at the *FOXP3* TSDR (Fig. 4B). The IL-6R^hi^TIGIT^−^ Treg population also showed 90.6% demethylation of a sequence in exon 2 of *CTLA4* (Fig. 4B), which was almost identical to IL-6R^hi^TIGIT^+^ (92.0%) and IL-6R^lo^TIGIT^+^ (89.0%) mTregs, but in marked contrast to memory Teff cells, in which only 37.4% of cells were demethylated at this region of *CTLA4* (Fig. 4B).

Next, we investigated whether the Treg subsets were anergic to TCR stimulation (anti-CD3/anti-CD28), when cultured *in vitro* in the absence or presence of a high dose of IL-2 (100 U/ml). Consistent with their Treg phenotypes, all three mTreg subsets, IL-6R^hi^TIGIT^−^, IL-6R^hi^TIGIT^+^ and IL-6R^lo^TIGIT^+^ mTregs were almost completely anergic to T cell stimulation, as compared to memory Teffs (Fig. 3 in Ref. [45]). In the presence of IL-2, and in contrast to the IL-6R^hi^TIGIT^+^ and IL-6R^lo^TIGIT^+^ mTregs, IL-6R^hi^TIGIT^−^ mTregs showed modest capacity to proliferate, although much less than conventional memory Teffs (Fig. 4C). The ability of a majority of IL-6R^hi^TIGIT^−^ mTregs to proliferate using stimulus conditions that are unable to induce a proliferative response from IL-6R^hi^TIGIT^+^ and IL-6R^lo^TIGIT^+^ mTregs suggests that Teffs are present in the IL-6R^hi^TIGIT^−^ mTregs and that they likely represent a portion of the FOXP3^−^ and possibly FOXP3^+^ cells in the population. It is likely that in the absence of IL-2, the lack of proliferation from the IL-6R^hi^TIGIT^−^ subset is due to the potent suppressive effect of the Tregs present in the same sorted population.

### 3.5 IL-6R^hi^TIGIT^−^ Tregs isolated *ex vivo* have a Th17 transcriptional profile

**Fig. 5:**
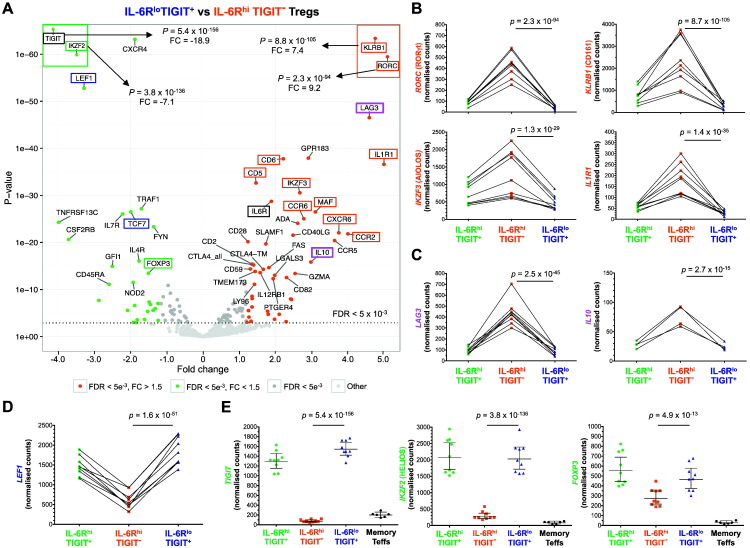
*Ex vivo* IL-6R^hi^TIGIT^−^ mTregs show a distinct Th17 transcriptional profile compared to conventional IL-6R^lo^TIGIT^+^ mTregs. (A) Volcano plot depicts the differential expression of 579 immune genes in IL-6R^hi^TIGIT^−^ and IL-6R^lo^TIGIT^+^ mTregs sorted *ex vivo* from nine healthy donors using NanoString. (B–D) Illustrative examples depicting the expression (GeoMean ± 95% CI) of (i) Th17 signature genes (marked in red), including *RORC* (RORγt), *KLRB1* (CD161), *IL1R1*, *IKZF3* (AIOLOS), and *CCR6* (B); (ii) Tr1 signature genes *LAG3* and *IL10* (marked in purple) (C); (iii) the transcription factors *LEF1* and *TCF7* (TCF1) (marked in blue), involved in the suppression of Th17 differentiation; and (iv) Treg signature genes (marked in green), including *TIGIT*, *HELIOS* and *FOXP3* (D), which were most differentially expressed in IL-6R^hi^TIGIT^−^ mTregs compared to their IL-6R^lo^TIGIT^+^ counterparts. Sorting markers used for the flow-sort purification of the assessed Treg subsets are marked in black. *P* values were calculated using two-tailed paired non-parametric Wilcoxon signed rank tests, comparing the normalised NanoString read counts between IL-6R^hi^TIGIT^−^ and IL-6R^lo^TIGIT^+^ mTregs.

To investigate the transcriptional profile of IL-6R^hi^TIGIT^−^ mTregs, we assessed the mRNA expression of 579 immune genes in sorted cells from nine healthy donors (Table 2 in Ref. [45]). The expression of TIGIT and IL-6R was assessed post-sorting and displayed a distinct expression in the respective subsets, indicating a very high level of purity (Fig. 1D in Ref. [45]). We found that *ex vivo* IL-6R^hi^TIGIT^−^ mTregs showed a distinct Th17 transcriptional signature (Fig. 5A), marked by the specific higher expression of the Th17 lineage discrimination transcription factor RORγt (encoded by *RORC*). The observed Th17 signature was also defined by higher expression of additional Th17 genes, including *KLRB1* (CD161), *CCR6*, *IL1R1* and *IKZF3* (AIOLOS) [48], [49], [50], [51], in IL-6R^hi^TIGIT^−^ mTregs (Fig. 5A,B). The results obtained at the transcriptional level were consistent with the results obtained at the protein level, as illustrated by the immunophenotyping of two Th17 signature surface receptors, CD161 and CCR6 (Fig. 5 in Ref. [45]). To investigate if the expression of IL-6R discriminates cells displaying a Th17 transcriptional signature within the TIGIT^−^ mTreg population, we compared the expression of these two canonical Th17 markers in both IL-6R^hi^ and IL-6R^−/int^ (corresponding to the lower 70th percentile of IL-6R expression in Tregs) TIGIT^−^ mTregs (Fig. 6A in Ref. [45]). The frequency of both CD161^+^ (44.0% *versus* 27.1%, *P* = 1.0 × 10^− 14^) and CCR6^+^ (78.8% *versus* 57.4%, *P* = 5.4 × 10^− 5^; Fig. 6B in Ref. [45]) cells were significantly increased in the IL-6R^hi^ subset, indicating that elevated IL-6R expression provides a better discrimination of Th17 Tregs (together with some Th17 Teffs) within the TIGIT^−^ compartment.

Furthermore, we also noted the increased expression of *LAG3* and *IL10* (Fig. 5C), which have been described as markers of the IL-10-producing FOXP3^−^ Tr1 subset [52], [53], and could also account for a proportion of the cells with a methylated *FOXP3* TSDR and a lack of FOXP3 expression in this population. Conversely, we found that the expression of two transcription factors, *LEF1* and *TCF7* (encoding TCF1), previously shown to co-ordinately repress the Th17 transcriptional program [54], [55], were specifically downregulated in IL-6R^hi^TIGIT^−^ mTregs (Fig. 5D). These data demonstrate a strong transcriptional commitment of IL-6R^hi^TIGIT^−^ mTregs to the Th17 phenotype. We observed a concomitant downregulation of the Treg genes *FOXP3* and *IKZF2* (HELIOS) (Fig. 5E), and an upregulation of *CTLA4* (Fig. 5A), which is consistent with the observed decreased frequency of FOXP3^+^ and HELIOS^+^ cells, but increased frequency of CTLA-4^+^ cells within IL-6R^hi^TIGIT^−^ mTregs (Fig. 3B,C). This downregulation of the Treg signature genes was particularly pronounced when comparing the IL-6R^hi^TIGIT^−^ to IL-6R^hi^TIGIT^+^ mTregs, which are a subset of highly activated Tregs, as evidenced by the elevated expression of HLA class II genes (Fig. 7 in Ref. [45]).

### 3.6 *In vitro* activated IL-6R^hi^TIGIT^−^ Tregs produce high levels of cytokines and increased expression of the pro-inflammatory IL-1β and IL-23 receptors

**Fig. 6:**
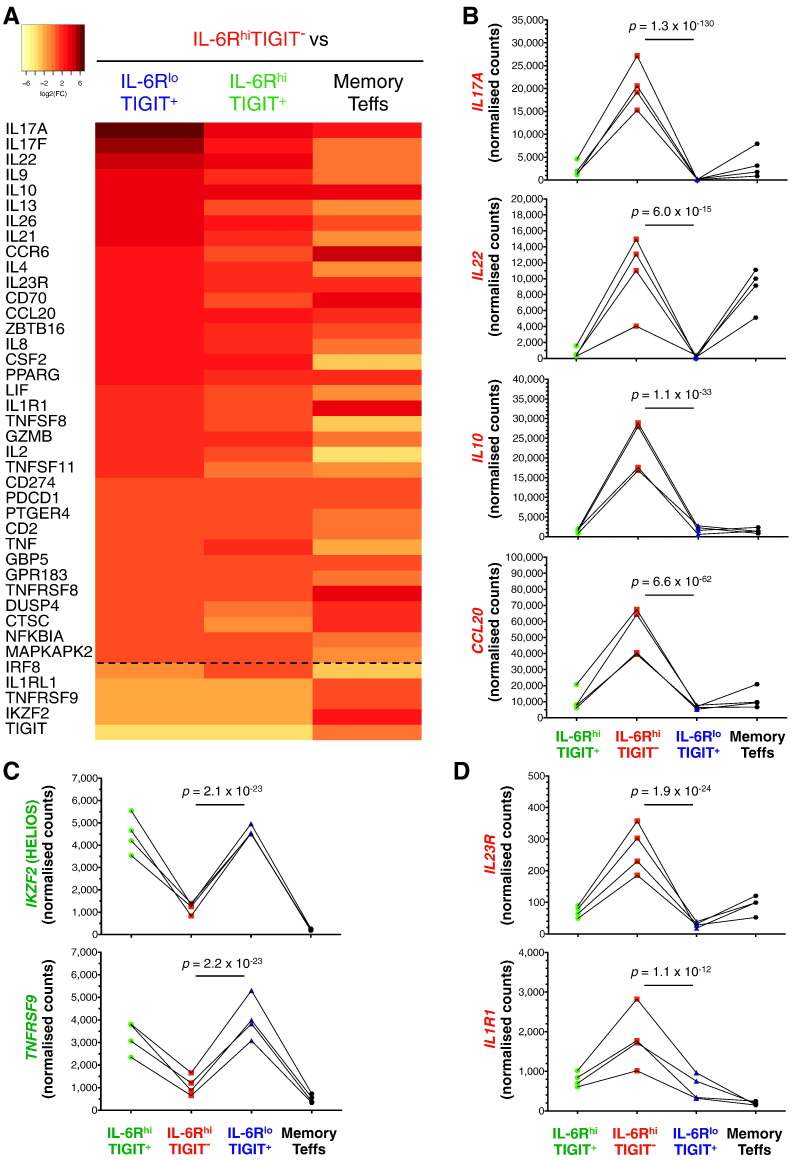
IL-6R^hi^TIGIT^−^ Tregs display a significant cytokine-producing potential upon *in vitro* activation. (A) Heatmap depicts the differential expression of 40 selected genes between sorted IL-6R^hi^TIGIT^−^ mTregs and: (i) IL-6R^lo^TIGIT^+^ mTregs; (ii) IL-6R^hi^TIGIT^+^ mTregs; or (iii) memory Teff cells from four healthy donors, upon *in vitro* activation with PMA + ionomycin. The genes shown in the figure represent the subset of 40 genes that were both: (i) significantly upregulated (adjusted *P* < 10^− 5^) in IL-6R^hi^TIGIT^−^ mTregs upon *in vitro* simulation; and (ii) differentially expressed (adjusted *P* < 10^− 4^) between activated IL-6R^hi^TIGIT^−^ and IL-6R^lo^TIGIT^+^ mTregs. (**B**) Illustrative examples of cytokine genes upregulated in activated IL-6R^hi^TIGIT^−^ mTregs, including both pro- (IL-17A, IL-22 and CCL20) and anti-inflammatory (IL-10) cytokines. (C) Illustrative examples depicting downregulated Treg signature genes in activated IL-6R^hi^TIGIT^−^ Tregs, including *IKZF2* (encoding for HELIOS) and *TNFRSF9*. (D) The expression of the IL-23 and IL-1 receptor genes (*IL23R* and *IL1R1*) were also specifically upregulated in activated IL-6R^hi^TIGIT^−^ mTregs.

To examine the effect of activation on the induction of the transcriptional programme of IL-6R^hi^TIGIT^−^ mTregs, we next assessed the expression of the 579 immune genes on sorted cells from the three Treg subsets from four healthy donors in response to *in vitro* stimulation with PMA and ionomycin (Table 3 in Ref. [45]). Consistent with their Th17 transcriptional profile, IL-6R^hi^TIGIT^−^ mTregs produced a large number of different cytokines, most notably Th17-specific cytokines, including IL-17, IL-22 and CCL20 (Fig. 6A,B). Notably, IL-6R^hi^TIGIT^−^ mTregs were also found to produce very high levels of the anti-inflammatory cytokine IL-10 (Fig. 6A,B). Importantly, the expression of genes that were specifically upregulated in IL-6R^hi^TIGIT^−^ mTregs were found to be remarkably similar to a set of signature genes that were uniquely expressed in a population of mouse lamina propria Tregs [56]. This set of genes included the expression of signature genes such as *CTLA4*, *LAG3*, *CCR2*, *CCR5*, *IRF4*, *MAF* and *IKZF3* (AIOLOS) *ex vivo* (Table 2 in Ref. [45]), and the expression of *IL10* and *GZMB* upon *in vitro* stimulation (Table 3 in Ref. [45]), which suggests that the IL-6R^hi^TIGIT^−^ mTregs we identify in circulation in humans share a similar origin to the murine intestinal Tregs.

*In vitro* stimulated IL-6R^hi^TIGIT^−^ mTregs produced higher levels of certain cytokines than even conventional CD45RA^−^ memory Teffs (*e.g.* 6.1-fold and 15.1-fold increase in the expression of *IL17A* and *IL10* compared to Teffs; Fig. 6A,B), whilst expressing lower levels of the Treg genes *IKZF2* (HELIOS) and *TNFRSF9* (CD137) (Fig. 6C). In addition to cytokine production, *in vitro* stimulation also induced the expression of *IL1R1* and *IL23R* on IL-6R^hi^TIGIT^−^ mTregs (Fig. 6D), suggesting an increased sensitivity to IL-1β and IL-23 signalling.

**Fig. 7:**
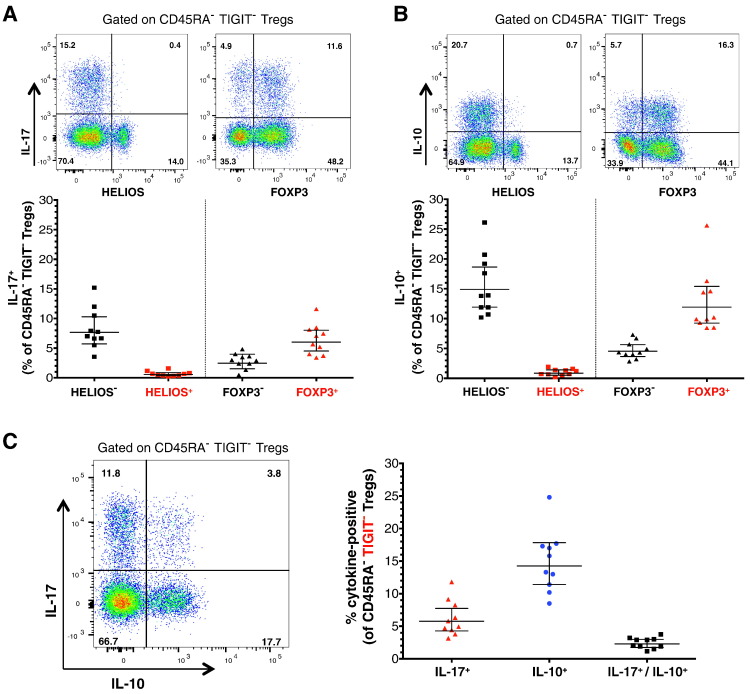
IL-17 and IL-10 are not produced by the same cells. (A,B) Data shown depict the frequency (GeoMean ± 95%CI) of IL-17^+^ (A) and IL-10^+^ (B) cells among CD45RA^−^ TIGIT^−^ mTregs, stratified by the expression of HELIOS and FOXP3. IL-17 and IL-10 production was assessed by intracellular flow cytometry in freshly isolated PBMCs from 10 healthy donors, following *in vitro* activation with PMA + ionomycin. (C) Data depict the frequency (GeoMean ± 95%CI) of IL-17 and IL-10 single-producers as well as IL-17/IL-10 double producers among the CD45RA^−^ TIGIT^−^ Treg subset.

To investigate the functional heterogeneity within IL-6R^hi^TIGIT^−^ mTregs, we then examined the production of IL-17 and IL-10 at the single-cell level by flow cytometry following *in vitro* activation. Given that IL-6R is shed upon *in vitro* activation, we could not use it to delineate IL-6R^hi^TIGIT^−^ mTregs, but instead focused on the total TIGIT^−^ mTreg population. Consistent with previous observations [57], [58], we showed that the HELIOS^−^ fraction of TIGIT^−^ mTregs contained the vast majority of cytokine-producing Tregs (Fig. 7A,B). In contrast, there was a much higher level of heterogeneity among the FOXP3-defined subsets, with both FOXP3^+^ and FOXP3^−^ Tregs showing the ability to produce IL-10 and IL-17 (Fig. 7A,B). We found that the majority of IL-10 and IL-17 producing cells were distinct (Fig. 7C), indicating that cellular heterogeneity among HELIOS^−^ Tregs is responsible for the production of these two very distinct cytokines observed in IL-6R^hi^TIGIT^−^ mTregs (Fig. 6B). In addition, we assessed the expression of RORγt by flow cytometry on a subset of 4 healthy donors. Of note, the frequency of RORγt^+^ cells was distinctly increased on IL-6R^hi^TIGIT^−^ mTregs (44.2%) as compared to TIGIT^−^ mTregs with lower expression of IL-6R (15.0%; Fig. 8A in Ref. [45]), indicating that elevated expression of IL-6R is critical for the delineation of the Th17 signature with TIGIT^−^ mTregs. Furthermore, in agreement with the observation that IL-17-producing cells are mainly FOXP3^+^, we also found that the frequency of FOXP3^+^ cells was strongly increased on RORγt^+^ (78.0%) as compared to their RORγt^−^ counterparts (51.7%) within IL-6R^hi^TIGIT^−^ mTregs, and was very similar to the frequency on TIGIT^+^ mTregs (83.4%; Fig. 8B in Ref. [45]).

### 3.7 IL-6R^hi^TIGIT^−^ Treg chemokine expression profile is consistent with a tissue-homing effector Treg subset

To assess the potential tissue-homing properties of IL-6R^hi^TIGIT^−^ mTregs, we next investigated their chemokine receptor profile *ex vivo* (Fig. 9A in Ref. [45]). Using the definition of the main Th cell lineages described previously [59], we confirmed the specific increase in the Th17 subset within IL-6R^hi^TIGIT^−^ mTregs, with a concomitant decrease in the frequencies of the Th2 and Th22 subsets compared to conventional TIGIT^+^ mTregs (Fig. 9B in Ref. [45]). In contrast, the frequency of the Th1 subset was not significantly altered in the different Treg populations (Fig. 9B in Ref. [45]). Analysis of the expression of the individual chemokine receptors also revealed that the profile of IL-6R^hi^TIGIT^−^ mTregs was more similar to TIGIT^+^ mTregs than to the TIGIT^−^ mTregs expressing lower levels of IL-6R (IL-6R^−/int^), which were more similar to memory Teff cells (Fig. 9C in Ref. [45]). Furthermore, we noted that the frequency of CCR4^+^ cells was particularly elevated within IL-6R^hi^TIGIT^−^ mTregs (96.8%); CCR4 has previously been described as a marker of highly suppressive, tissue-infiltrating, effector Tregs [60]. Similarly, we also observed that the frequency of another receptor recently shown to mark the more suppressive effector FOXP3^+^ Tregs, CD15s [61], was much higher for IL-6R^hi^TIGIT^−^ mTregs (46.8%), compared with IL-6R^−/int^ mTregs (15.8%).

Transcriptional analysis also identified the increased expression of two additional chemokine receptors, CCR2 and CCR5, among the top differentially expressed genes in *ex vivo* isolated IL-6R^hi^TIGIT^−^ mTregs (Table 2 in Ref. [45]). To investigate the gut-homing capacity of IL-6R^hi^TIGIT^−^ mTregs, we also assessed the expression of integrins α4 and β7 associated with migration to the colon, and the small intestine homing marker CCR9 (Fig. 10A,B in Ref. [45]). Expression of both the α4 monomer and the α4β7 heterodimer as well as CCR9, were increased on IL-6R^hi^TIGIT^−^ mTregs (Fig. 10C in Ref. [45]), demonstrating that a portion of IL-6R^hi^TIGIT^−^ mTregs possess the capacity to migrate to the gut.

## 4 Discussion

The critical role of Tregs in mediating protection from autoimmunity has led to many studies over the past decades, which have vastly contributed to our knowledge about this immune subset, and to the development of therapeutic strategies targeting Tregs. However, with the development of novel genomics [46] and proteomics tools [62], it is becoming apparent that there is phenotypic and functional heterogeneity among Tregs, which could lead to unexpected outcomes in clinical trials targeting Tregs in transplantation, tumour therapy and in autoimmune diseases.

In the present study we performed in-depth immunophenotyping of the expression of IL-6R on the human CD4^+^ CD127^low^CD25^+^ T cell compartment *ex vivo*. Although IL-6R has been shown to be expressed by human Tregs [35], [63], there is limited data describing its expression on different Treg subsets, and there has been no attempt to characterise the function of Tregs stratified by IL-6R expression. This is due to a combination of factors, namely the limited range of expression of IL-6R on human T cells, the large genetically-regulated inter-individual variation in expression levels [38], the high sensitivity of IL-6R to shedding in response to cell stress or activation [64] and an incomplete optimisation of the antibodies and cytometric fluorescent labels to reach the greatest sensitivity of detection of the receptor, all of which hamper the precise quantitative measurement of this receptor by flow cytometry in humans. Our results therefore provide the first detailed phenotypic and functional characterisation of IL-6R-expressing CD127^low^CD25^+^ T cell subsets in humans, and support a role of IL-6 signalling on the stability and function of differentiated Treg subsets.

We identified a subset of circulating IL-6R^hi^TIGIT^−^ CD127^low^CD25^+^ T cells (designated as IL-6R^hi^TIGIT^−^ mTregs) with a Th17 transcriptional profile, marked by the expression of the RORγt transcription factor, high CTLA-4 expression, and the capacity to produce high levels of both pro- and anti-inflammatory cytokines, upon *in vitro* stimulation (summarised in Table 1). Although we note that a population of Th17 Tregs has been previously described in humans [19], [20], [21], [22], our data refines the characterisation of this subset, and reveals a remarkably consistent Th17 transcriptional profile and the capacity to produce a wide range of cytokines, most notably Th17-type cytokines, upon *in vitro* stimulation.

A key finding was the observation that IL-6R^hi^TIGIT^−^ mTregs are extremely effective at suppressing the proliferation of autologous Teffs *in vitro*, even more so than IL-6R^hi^TIGIT^+^ mTregs, which exhibit all the classic hallmarks of conventional suppressive Tregs. This observation is even more remarkable if we consider that a proportion of the IL-6R^hi^TIGIT^−^ mTreg population consists of non-anergic FOXP3^−^ memory Teffs. A limitation of the current study is that, owing to the cellular heterogeneity of the IL-6R^hi^TIGIT^−^ CD127^low^CD25^+^ T cell population, we cannot exclude the possibility that the Th17 transcriptional signature is caused by a subset of CD127^low^CD25^+^ Th17 Teff cells. The observation that the majority of IL-17-producing cells, as well as RORγt^+^ IL-6R^hi^TIGIT^−^ mTregs, are present in the FOXP3^+^ compartment supports the regulatory nature of IL-6R^hi^TIGIT^−^ mTregs displaying a Th17 profile, as illustrated by the good correlation that we observed between the frequency of FOXP3^+^ cells and the frequency of *FOXP3* TSDR demethylation. However, in this study we were not able to determine whether the IL-17 secreting RORγt^+^ FOXP3^+^ T cells display a demethylated TSDR. Therefore, we cannot formally exclude the possibility that IL-17-secreting RORγt^+^ FOXP3^+^ cells represent a subset of activated memory Teffs with a methylated TSDR, and account for the difference in the frequency between FOXP3^+^ cells at the protein level and the frequency of TSDR demethylated cells within IL-6R^hi^TIGIT^−^ mTregs.

In addition, IL-6R^hi^TIGIT^−^ mTregs were found to produce very high levels of the anti-inflammatory cytokine IL-10, which is a key mediator of Treg suppression *in vivo*, suggesting the possibility that a small proportion of Tr1 Tregs present in the FOXP3^−^ fraction of the IL-6R^hi^TIGIT^−^ mTreg population could account for the increased suppressive capacity of this population, through the production of high levels of IL-10. Further work will be necessary to dissect the cellular and functional heterogeneity of this population at the single-cell level, and to investigate the TSDR demethylation of the cytokine-producing subsets. Despite the cellular heterogeneity of IL-6R^hi^TIGIT^−^ mTregs, our findings have important implications for the clinical studies exploring the expansion of CD4^+^ CD127^low^CD25^+^ T cells for the treatment of autoimmune diseases, and identify a population of IL-6R^hi^TIGIT^−^ T cells within this compartment containing a significant fraction of activated effector Tregs, which likely mediate their suppression in part by IL-10 production [65] and CTLA-4 binding to the co-stimulatory molecules CD80 and CD86 [66], [67]. Furthermore, the elevated expression of CD25 compared to memory Teff cells, combined with the high sensitivity to IL-2 *in vivo* suggests that IL-6R^hi^TIGIT^−^ mTregs are also able to mediate their suppression mechanism through IL-2 consumption [65].

These results are consistent with a previous study, characterising a subset of HELIOS^−^ mTregs marked by the expression of the IL-1R and the transcription factor AIOLOS in humans, which were found to be more suppressive than conventional HELIOS^+^ Tregs *ex vivo* [58]. In that study addition of exogenous IL-1β abrogated their suppressive capacity, through a mechanism dependent on the expression of the IL-1R [58]. Furthermore, another study has described a subset of FOXP3^+^ Tregs marked by the expression of CD161 with high *in vitro* suppressive capacity [68]. Similarly to the IL-6R^hi^TIGIT^−^ mTregs described here, these CD161^+^ FOXP3^+^ Tregs also displayed the capacity to produce IL-17 following *in vitro* activation, and contained predominantly cells demethylated at the *FOXP3* TSDR [68]. In contrast to the data from Pesenacker *et al*., we characterise a more purified subset of HELIOS^−^ IL-6R^hi^TIGIT^−^ mTregs, and use a more quantitative method [46] to assess the exact frequency of demethylated cells in this subset. The identification of a suppressive HELIOS^−^ TIGIT^−^ mTreg population is particularly striking given that TIGIT^+^ HELIOS^+^ Tregs are traditionally thought to harbour the most suppressive Treg subset [69], [70], [71]. However, we note in two of these studies [69], [71] that naive Tregs were not excluded from the sorted TIGIT^−^ Tregs used in the suppression assays and, therefore, may have diluted the suppressive capacity, when considered on a per cell basis, of the mTregs within the sorted TIGIT^−^ Treg population.

From an evolutionary perspective, there is evidence pointing to a common developmental pathway in the differentiation of both induced-Treg and Th17 lineages [27], suggesting that Th17 Tregs could play an important role in the regulation of the immune responses and commensal bacteria composition in the gut [72]. This is consistent with the recent data showing that the composition of intestinal bacterial commensals is critical in regulating the frequency of a highly suppressive subset of RORγt^+^ Tregs in mice [23], [24], which are thought to regulate the interplay between commensal and pathogenic bacteria and the host immune system in the gut, while preventing chronic inflammation. Furthermore, another recent study in mice has shown that RORγt^+^ FOXP3^+^ cells represent a stable Treg lineage with epigenetic marks of conventional RORγt^−^ FOXP3^+^ Tregs, and are potent suppressors of inflammation in a colitis model [25]. The transcriptional profile of these murine RORγt^+^ FOXP3^+^ Tregs is very similar to the human IL-6R^hi^TIGIT^−^ mTregs described in our study, including the expression of Th17 signature genes but also of Treg effector genes such as *IL10* and *CTLA4*.

Our findings suggest that a similar gut-resident T cell subset exists in humans [73], which is mobilised and expanded by IL-2 treatment and consequently becomes more detectable in the blood. In agreement with this hypothesis, IL-6R^hi^TIGIT^−^ mTregs displayed a remarkably similar transcriptional profile with a population of Tregs resident in the mucosal tissues [56], which strongly supports that IL-6R^hi^TIGIT^−^ mTrsgs are located primarily in the intestine and mesenteric lymph nodes. In agreement with this hypothesis, IL-6R^hi^TIGIT^−^ mTregs displayed a chemokine receptor profile consistent with a tissue-homing T cell subset, including the elevated expression of markers such as CCR2, CCR4, CCR5 and CCR6 and the adhesion marker CD15s, as well as the specific upregulation of the prototypical small intestine (CCR9) and colon (α4β7) homing receptors. These data demonstrate the tissue-homing properties of IL-6R^hi^TIGIT^−^ mTregs, and their capacity to migrate to the gut, therefore supporting their potential intestinal nature, which is similar to their murine intestinal RORγt^+^ FOXP3^+^ Treg analogues. Although the expansion of Tregs in circulation in response to IL-2 is not restricted to the IL-6R^hi^TIGIT^−^ subset, we hypothesise that this subset could be particularly relevant for regulating Th17 responses, and expansion and trafficking in response to IL-2 signalling by IL-6R^hi^TIGIT^−^ cells suggest an important role in the regulation of tissue inflammation and autoimmune reactions. One possibility that has been suggested in a mouse model is that IL-10 production by FOXP3^+^ Tregs is key to promote equilibrium with pro-inflammatory IL-17-producing RORγt^+^ Th17 effector cells [74]. Our observation that IL-10 is expressed at high levels by IL-6R^hi^TIGIT^−^ mTregs, suggests that they represent a major IL-10-producing Treg subset in humans, and play a critical role in the regulation of the IL-17-mediated immune responses at the sites of infection, which is key to maintain the homeostasis between commensal bacteria and invading pathogens at mucosal barrier surfaces.

In the context of human autoimmune disease, IL-17-producing FOXP3^+^ Tregs with *in vitro* suppressive capacity have been shown to be recruited to the intestinal mucosa in active Crohn's disease patients [75], to the inflamed joints of juvenile idiopathic arthritis (JIA) patients [68] and to peripheral blood of rheumatoid arthritis patients [76]. In addition, in psoriasis patients, IL-6R-expressing Tregs have been previously shown to be recruited to the inflamed skin, where they co-localise with the pathogenic IL-17 producing Th17 effector cells [35]. These observations are consistent with the recruitment of activated Th17-signature positive Tregs with suppressive function to the sites of infection to prevent or limit Th17 effector tissue damage. An intriguing question that remains to be addressed is the potential effect of prolonged exposure to pro-inflammatory signals on the differentiation and function of these RORγt^+^ FOXP3^+^ Tregs, particularly in the setting of chronic inflammation. We have shown that mTregs can signal through the IL-6R by activating pSTAT3, and have previously demonstrated that elevated expression of IL-6R on CD4^+^ T cells translates to increased IL-6 signalling [38]. Furthermore, IL-6 is known to relieve the FOXP3-mediated suppression of RORγt [30], leading to the disruption of the balance of RORγt and FOXP3 expression in IL-6R^hi^TIGIT^−^ mTregs, and to the concomitant instability of FOXP3 expression in these cells. We therefore hypothesise that in genetically-susceptible individuals at the *IL6R* locus, the increased IL-6 signalling potential increases the sensitivity of IL-6R^hi^TIGIT^−^ mTregs to IL-6, resulting in the loss of regulatory potential and to the trans-differentiation into pathogenic IL-17-producing ex-Tregs [77], [78], [79].

In summary, our findings identify a subset of HELIOS^−^ FOXP3^+^ Tregs, which can be detected *in vivo* based on the expression of two surface markers, IL-6R and TIGIT, and show that subcutaneously administered IL-2 can promote the expansion and trafficking of these cell subsets into circulation. Although the frequency of these cells is usually low in peripheral blood, ranging from 0.4% to 1.4% of total circulating CD45RA^−^ memory CD4^+^ T cells, their frequency is expanded with single doses of IL-2 of around 400,000 IU/m^2^ [15]. Moreover, the strong *in vitro* suppressive capacity of IL-6R^hi^TIGIT^−^ mTregs coupled with their potential to produce a diversity of pro- and anti-inflammatory cytokines, suggests that their frequency should be carefully monitored, particularly in autoinflammatory diseases, in which chronic inflammation could promote the migration of these cells from the gut to inflamed tissues. As clinical studies aiming to expand Tregs become more prevalent, another important application of these markers will be to assess preferential expansion of specific Treg and additional CD127^low^CD25^+^ Teff subsets, and inform on disease-specific dosing and patient selection, especially in conditions where IL-17 is a cause of tissue damage [80]. Our findings also suggest a biological mechanism underpinning the genetic association of the *IL6R* with human inflammatory diseases, whereby in genetically susceptible individuals, increased IL-6 signalling could impair the regulatory function of a tissue-resident Treg subset with potent suppressive potential. These data provide a rationale for specific targeting of this molecular pathway in diseases genetically associated with the *IL6R* locus, rather than non-specific blockade of IL-6 signalling which brings with it an increased risk of infection that might not be acceptable in the context of T1D in children.

## Funding

This research was supported by the JDRF (9-2011-253/5-SRA-2015-130-A-N), the Wellcome Trust (WT091157/107212 and WT083650/Z/07/Z), the National Institute for Health Research (NIHR) Cambridge Biomedical Research Centre, and the Cambridge Clinical Trials Unit (CCTU).

RCF is funded by a JDRF advanced post-doctoral fellowship (3-APF-2015-88-A-N).

## Author contributions

RCF, LSW and JAT conceived the study, designed research and wrote the paper; RCF, DBR, MLP, LP and JJO performed research; RCF and ARG analysed the data; FWL, LSW and JAT designed and coordinated the DILT1D mechanistic study and patient recruitment.

## Competing interests

The authors have no conflicting financial interests.
